# Different Subpopulations of Developing Thymocytes are Associated with Adherent (Macrophage) or Nonadherent (Dendritic) Thymic Rosettes

**DOI:** 10.1155/1991/49025

**Published:** 1991

**Authors:** Ken Shortman, David Vremec

**Affiliations:** The Walter and Eliza Hall Institute of Medical Research Post Office Royal Melbourne Hospital Victoria 3050 Australia

## Abstract

Thymic rosettes (ROS), structures consisting of thymic lymphoid cells attached to a central stromal cell, were isolated from mouse thymus by collagenase digestion and unit-gravity elutriation. The ROS were then separated into those where the stromal cells were either macrophage-like (M-ROS) or dendritic cell-like (D-ROS), on the basis of the differences in adherence properties or in the level of MAC-1 surface antigen. The ROS were then dissociated and the thymocyte content analyzed by immunofluorescent staining and flow cytometry. M-ROS and D-ROS differed in thymocyte composition, although the major component of both was the CD4^+^CD8^+^ cortical thymocyte. D-ROS were enriched in thymocytes expressing high levels of surface T-cell antigen receptor (TcR) and the associated CD3 complex, and these included both CD4^+^CD8^+^CD3^+^^+^ and CD4^-^CD8^+^CD3^+^^+^ mature thymocytes. M-ROS were enriched in CD4^-^CD8^-^ thymocytes and had a reduced content of thymocytes expressing high TcR-CD3 levels; they nevertheless contained some mature thymocytes, but only of the CD4^+^CD8^-^CD3^+^^+^ category. Several lines of evidence indicated that the mature thymocytes in ROS were cells recently formed in the cortex, and were not from the medullary pool. ROSassociated mature thymocytes expressed lower levels of H-2K than free, mature thymocytes. The CD4^+^CD8^+^CD3^+^^+^ subpopulation, believed to be a developmental intermediate between cortical thymocytes and mature T cells, was present in both ROS populations. Further, late intermediates leading to both mature T-cell categories were evident in D-ROS, but only those leading to CD4^+^CD8^-^CD3^+^^+^ T cells were evident in M-ROS. The results are compatible with a role for ROS in TcR-specificity selection and in the final maturation steps in the thymic cortex.


*Developmental Immunology*, 1991, Vol. 1, pp. 225–235
Reprints available directly from the publisher
Photocopying permitted by license only

© 1991 Harwood Academic Publishers GmbH
Printed in the United Kingdom

### Different Subpopulations of Developing Thymocytes are Associated with Adherent (Macrophage) or Nonadherent (Dendritic) Thymic Rosettes

KEN SHORTMAN*† and DAVID VREMEC†

*†The Walter and Eliza Hall Institute of Medical Research, Melbourne, Australia*

Thymic rosettes (ROS), structures consisting of thymic lymphoid cells attached to a central stromal cell, were isolated from mouse thymus by collagenase digestion and unit-gravity elutriation. The ROS were then separated into those where the stromal cells were either macrophage-like (M-ROS) or dendritic cell-like (D-ROS), on the basis of the differences in adherence properties or in the level of MAC-1 surface antigen. The ROS were then dissociated and the thymocyte content analyzed by immunofluorescent staining and flow cytometry. M-ROS and D-ROS differed in thymocyte composition, although the major component of both was the $CD4^+CD8^+$ cortical thymocyte. D-ROS were enriched in thymocytes expressing high levels of surface T-cell antigen receptor (TcR) and the associated CD3 complex, and these included both $CD4^+CD8^-CD3^{++}$ and $CD4^-CD8^+CD3^{++}$ mature thymocytes. M-ROS were enriched in $CD4^-CD8^-$ thymocytes and had a reduced content of thymocytes expressing high TcR–CD3 levels; they nevertheless contained some mature thymocytes, but only of the $CD4^+CD8^-CD3^{++}$ category. Several lines of evidence indicated that the mature thymocytes in ROS were cells recently formed in the cortex, and were not from the medullary pool. ROS-associated mature thymocytes expressed lower levels of H-2K than free, mature thymocytes. The $CD4^+CD8^+CD3^{++}$ subpopulation, believed to be a developmental intermediate between cortical thymocytes and mature T cells, was present in both ROS populations. Further, late intermediates leading to both mature T-cell categories were evident in D-ROS, but only those leading to $CD4^+CD8^-CD3^{++}$ T cells were evident in M-ROS. The results are compatible with a role for ROS in TcR-specificity selection and in the final maturation steps in the thymic cortex.

KEYWORDS: Thymic macrophages, thymic dendritic cells, thymic stromal cells, thymocyte maturation.

### INTRODUCTION

The stromal cells of the thymus are believed to dictate several phases of T-lymphocyte development. Their role includes promotion of proliferation and differentiation at early stages, before TcR expression, and mediation of positive and negative selection of the specificity repertoire at later stages, after the TcR is expressed on the cell surface. One way of studying the interaction between developing T cells and the thymic stroma is to isolate from the thymus the interaction complexes between thymocytes and stromal cells. Kyewski and colleagues (Kyewski et al., 1982, 1987, 1989; Kyewski, 1987) have isolated preexistent structures termed thymic rosettes (ROS), consisting of a central stromal cell surrounded by adherent thymocytes. The central stromal cell has been reported to be either a macrophage (M-ROS) or a dendritic cell (D-ROS) (Kyewski et al., 1982), and these two forms of ROS are separable based on differences in adherence properties (Kyewski et al., 1987). They are also considered to have different functions and to have different locations within the thymus, M-ROS in the cortex, D-ROS in the medulla, or at the corticomedullary junction (Kyewski et al., 1982, 1987, 1989; Kyewski, 1987).

In a previous study (Shortman et al., 1989), we developed a single-step unit-gravity elutriation

*Corresponding author. Present address: The Walter and Eliza Hall Institute of Medical Research, Post Office Royal Melbourne Hospital, Victoria 3050, Australia.

procedure for the isolation of pure rosettes from collagenase digests of thymic stroma. We presented evidence that these represented the preexistent complexes, without significant cell exchange during isolation. The surface antigenic phenotype of the thymocytes associated with these isolated ROS appeared to be similar to that of the total free thymocytes, all main thymocyte subpopulations including mature cells being present. We now separate the rosettes into M-ROS and D-ROS fractions and find differences between them in the surface antigenic phenotype of the bound thymocytes. Detailed phenotypic analysis suggests that the mature cells present in both these structures represent cells recently formed in the cortex.

### RESULTS

### Separation of M-ROS and D-ROS

The pooled large ROS fraction from the elutriation procedure yielded about $10^5$ ROS per thymus, and these yielded a mean of $1.1 \times 10^6$ thymocytes if dissociated with EDTA. Several procedures were tested for the separation of putative M-ROS and D-ROS. The simplest and most effective procedure was according to differences in adherence properties, using an approach similar to that of Kyewski et al. (1987), but with the incubation time reduced to 50 min at 37°. Under these conditions, an average of 29% of all ROS-associated thymocytes were recovered from the adherent fraction (M-ROS) and 71% from the nonadherent (D-ROS) fraction. The ROS were sufficiently well spaced apart on the plastic surface during the adherence separation for interrosette exchange of thymocytes to be unlikely, but any thymocytes released from the adherent ROS during incubation would appear in the nonadherent fraction, so contaminating this group. Accordingly, other approaches to separating the two rosette classes were tested. Coating the ROS with anti-MAC-1 mAb and then separation with anti-Ig-coupled magnetic beads produced a separation into a group of "MAC-1$^{+}$" M-ROS and "MAC-1$^{-}$" D-ROS; 26% of ROS-associated thymocytes were recovered from the MAC-1$^{+}$ fraction, 74% from the MAC-1$^{-}$ fraction. This degree of separation was constant over a range of anti-MAC-1 mAb levels; however, we have not established whether the dendritic cells of D-ROS were totally MAC-1 negative, or merely expressed lower levels or less accessible surface MAC-1. This procedure had the advantage of avoiding any incubation at 37°, but required two centrifugation steps, a maneuver that we have found to cause some exchange of thymocytes between ROS (Shortman et al., 1989), and so might produce some cross-contamination of both ROS fractions. In all subsequent studies, we have used the adherence procedure as the primary method for separation of the putative M-ROS and D-ROS, and used the anti-MAC-1 and magnetic-bead approach only to check the main findings. To date, both procedures have given similar results.

### Intrathymic Localization of M-ROS and D-ROS

To determine if M-ROS and D-ROS were of different intrathymic location, intact thymuses were labeled with Hoechst 33342 by the transcapsular route, the M-ROS and D-ROS then isolated, and the associated thymocytes analyzed for the proportion of blue fluorescent cells. With a gate set so 4% of free thymocytes registered as blue fluorescent, 7.5% of M-ROS-associated thymocytes were positive, as opposed to 2.0% of D-ROS-associated thymocytes. With a less stringent gate, where 6% of free thymocytes registered blue fluorescent, the values were 9.5% for M-ROS and 3.5% for D-ROS. This indicates the M-ROS were relatively frequent in the outer cortex, but that D-ROS were infrequent in this zone and so mainly located further within the thymus. However, this discrimination was not as sharp as that obtained by comparing a population known to be enriched in the outer cortex (e.g., CD4$^{+}$CD8$^{+}$ blasts) with a population known to be predominantly medullary (e.g., CD4$^{-}$CD8$^{+}$ and CD4$^{+}$CD8$^{-}$ thymocytes) (Scollay and Shortman, 1985).

### Expression of the TcR-CD3 Complex by Thymocytes Associated with M-ROS and D-ROS

Thymocytes can be divided into three distinct groups according to expression of the TcR-CD3 complex, as shown in Fig. 1, using an anti-CD3 stain. The three fluorescent peaks obtained represent cells expressing no (or extremely low) levels of TcR-CD3 (CD3$^{-}$), those expressing low but quite definite levels (CD3$^{+}$), and those expressing the high levels characteristic of peripheral T cells ($CD3^{++}$). Almost identical results are obtained by staining with an antibody against the TcR$\alpha\beta$ (data not shown). The $CD3^-$ and $CD3^+$ peaks represent predominantly $CD4^+CD8^+$ cortical thymocytes and TcR-$CD3^{++}$ peak represents predominantly $CD4^+CD8^-$ and $CD4^-CD8^+$ mature medullary thymocytes. We have previously shown (Shortman et al., 1989) that ROS-associated thymocytes in general give a similar pattern, but with some enrichment of cells bearing high levels of the TcR-CD3 complex; this finding is confirmed in Fig. 1. However, this picture for the total ROS fraction changed significantly when D-ROS and M-ROS were separated on the basis of adherence properties. Most of the $CD3^{++}$ cells were concentrated in the D-ROS, although it was clear that this fraction also contained $CD3^+$ and $CD3^-$ cells. The M-ROS had a much lower content of $CD3^{++}$ cells. Results obtained with C57BL/6 mice (not shown) were similar to those obtained with the CBA mice of Fig. 1, except that the proportion of $CD3^{++}$ cells amongst free thymocytes and amongst ROS-associated thymocytes was somewhat lower overall.

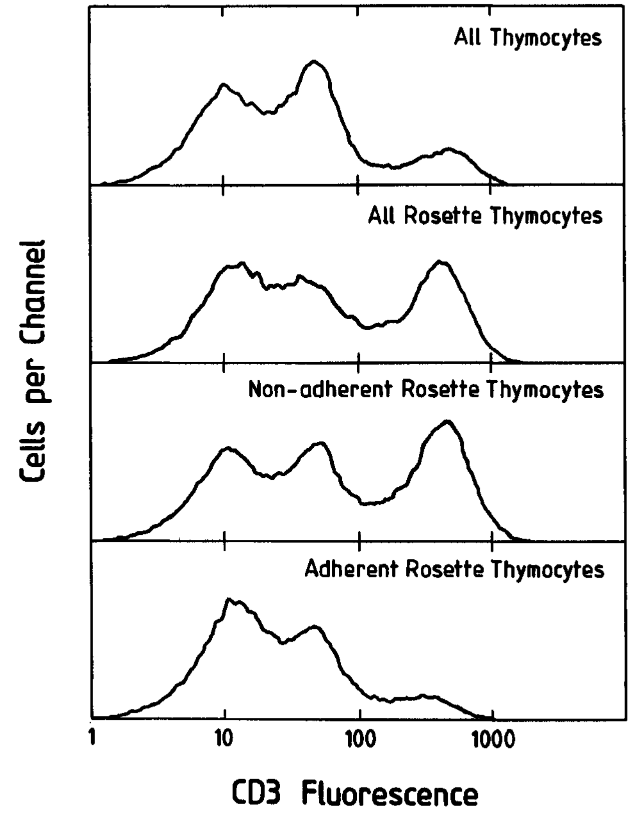

FIGURE 1 The distribution of thymocytes bearing different levels of the TcR-CD3 complex in ROS. "All thymocytes" represents a mechanically dissociated free thymocyte suspension; "nonadherent rosette thymocytes" were the dissociated cells from the D-ROS preparation, and "adherent rosette thymocytes" the dissociated cells from the M-ROS preparation. Most of the larger-sized stromal cells were gated out of the analysis on the basis of high low-angle light scatter and high side scatter. The cells were stained with biotinylated KT3-1.1 monoclonal antibody and PE–avidin second stage. The three peaks represent cells expressing negative to very low levels of CD3 ($CD3^-$), low levels of CD3 ($CD3^+$), and high levels of CD3 ($CD3^{++}$).

### Expression of CD4 and CD8 by Thymocytes Associated with M-ROS and D-ROS

In our earlier study (Shortman et al., 1989), we demonstrated that ROS included all four thymic populations defined by CD4 and CD8 expression, the dominant population being the small $CD4^+CD8^+$ cortical-type thymocyte. However, this disappointing similarity to thymocytes overall changed when M-ROS and D-ROS were separated (Fig. 2; Table 1), although $CD4^+CD8^+$ thymocytes remained the dominant cell in both ROS populations. Both $CD4^-CD8^+$ and $CD4^+CD8^-$ single positive thymocytes were at a higher level in the D-ROS fraction, in line with the enhanced level of $CD3^{++}$ cells in Fig. 1. The $CD4^-CD8^-$ population was at higher levels in the M-ROS fraction, in line with the proposed cortical location. However, a surprising finding was the association with M-ROS of an elevated level of $CD4^+CD8^-$ thymocytes, but not of $CD4^-CD8^+$ thymocytes (Fig. 2; Table 1). These findings, based on separation of ROS by adherence differences, were checked using the anti-MAC-1 and magnetic-bead separation procedures, with almost identical results. The findings were also checked with the C57BL/6 mouse strain, which has a higher cortical to medullary ratio and a correspondingly higher double positive to single positive ratio. The results were basically the same, except the $CD4^+CD8^-$ population of M-ROS, although present, did not stand out so clearly on contour plots, because of the dominance of $CD4^+CD8^+$ and $CD4^-CD8^-$ cells.

### Does the Composition of M-ROS Reflect that of the Outer Cortex?

It was possible that the unusual spectrum of cells associated with M-ROS simply reflected the com-

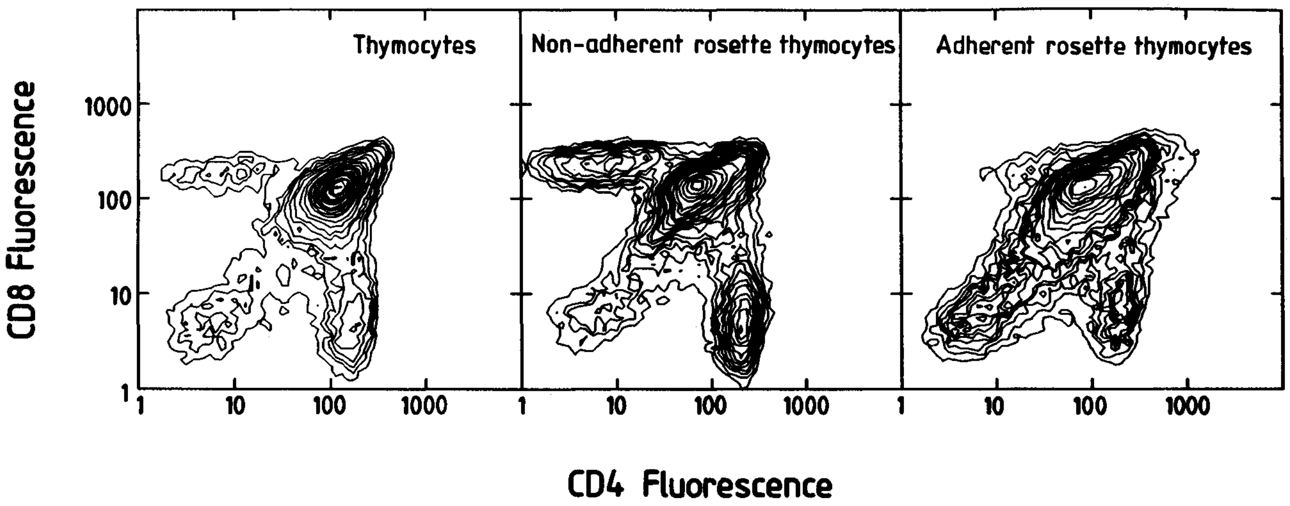

FIGURE 2 The expression of CD4 and CD8 by thymocytes associated with D-ROS and M-ROS. The analysis of the pooled ROS fraction for these markers has been presented in detail previously (Shortman et al., 1989). In this experiment, the D-ROS were separated as nonadherent rosettes, and the M-ROS as adherent rosettes. Similar results were obtained when the basis of separation was MAC-1$^-$ versus MAC-1$^+$ rosettes, using a magnetic-bead separation procedure.

position of the outer cortical region where the M-ROS were located. To test this, outer cortical cells were selectively stained with Hoechst 33342 by the transcapsular route, the thymus mechanically disrupted, and the free thymocytes stained for CD4 and CD8 expression. By gating for the 5% blue fluorescent cells, the distribution of the main thymocyte groups in the outer cortex was determined (Fig. 3). Outer cortical thymocytes were predominantly CD4$^+$CD8$^+$ and CD4$^-$CD8$^-$. Some CD4$^+$CD8$^-$ thymocytes could be detected in most preparations, but these were at a low level, always much less than the level of CD4$^-$CD8$^-$ cells. Thus, the composition of M-ROS differed both from that of the total thymus and from that of the outer cortex.

### Are the Single Positive Thymocytes of ROS Mature CD3$^{++}$ Cells?

Single positive thymocytes (CD4$^+$CD8$^-$ and CD4$^-$CD8$^+$) include not only the mature CD3$^{++}$ cells, mainly located in the medulla, but also immature CD3$^-$ cortical cells, which represent transit populations between CD4$^-$CD8$^-$ and CD4$^+$CD8$^+$ thymocytes. These "immature single positives" are found amongst both CD4$^-$CD8$^+$ thymocytes (Crispe and Bevan, 1987; MacDonald et al., 1988; Shortman et al., 1988) and CD4$^+$CD8$^-$ thymocytes (Matsumoto et al., 1989; Hugo et al., 1990), and so the single positives present in ROS might have been of this type. To distinguish mature from immature single positives, the ROS

TABLE 1
CD4- and CD8-Defined Thymus Populations Amongst ROS[a]

| Sample | Percent of total (mean±SEM) | | | | Ratio CD4$^+$CD8$^-$ / CD4$^-$CD8$^+$ |
|---|---|---|---|---|---|
| | CD4$^+$CD8$^-$ | CD4$^-$CD8$^+$ | CD4$^-$CD8$^-$ | CD4$^+$CD8$^+$ | |
| All free thymocytes | 4.9±0.9 | 1.9±0.3 | 2.0±0.3 | 91.2±0.7 | 2.6 |
| All ROS thymocytes | 6.2±0.4 | 2.6±0.7 | 4.2±1.0 | 87.0±2.3 | 2.4 |
| D-ROS thymocytes | 7.7±0.3 | 3.9±0.6 | 5.0±0.8 | 83.4±1.8 | 2.0 |
| M-ROS thymocytes | 6.8±0.9 | 1.8±0.2 | 8.1±2.7 | 83.3±2.1 | 3.8 |

[a]The results are pooled from five experiments resembling that of Figure 2, four where putative D-ROS and M-ROS were separated on the basis of adherence differences, and one where separation was based on MAC-1 expression. All experiments gave basically similar results. Note that the broad quadrants counted do not show as clear a distinction as the contour lines of Figure 1, because of the presence of a scatter of cells with atypical levels of CD4 and CD8; many of these were CD3$^-$, immature single positives.

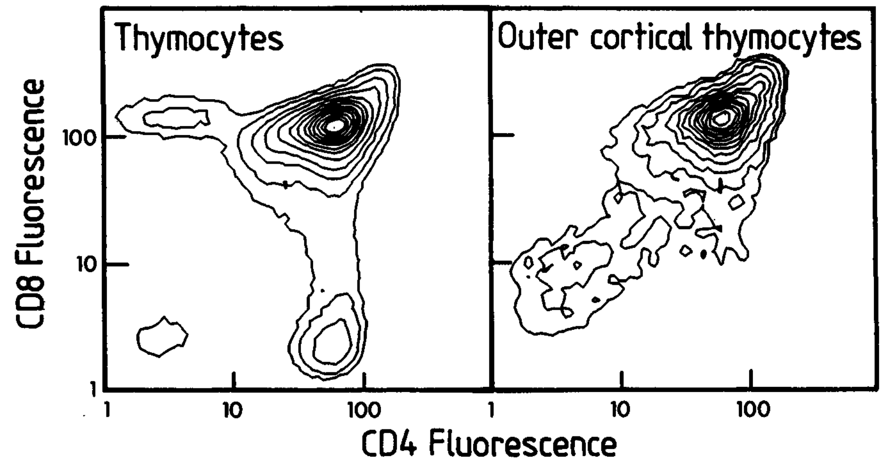

FIGURE 3 The expression of CD4 and CD8 by thymocytes in the outer cortex. Intact thymuses were dipped in Hoechst 33342 solution, so the cells in the subcapsular zone were selectively labeled. The thymuses were then disrupted, and the thymocytes immunofluorescent stained for CD4 and CD8 expression. The outer cortical thymocyte distribution was then obtained by three-color flow cytometry, gating for the 5% blue fluorescent cells.

thymocytes were stained in three fluorescent colors for CD4, CD8, and CD3, and the CD3 distribution on the gated single positives determined, as shown in Fig. 4.

The presence of both mature $CD3^{++}$ and immature $CD3^-$ cells was apparent within both the $CD4^-CD8^+$ and the $CD4^+CD8^-$ populations of free thymocytes. The single positives associated with the nonadherent D-ROS were predominantly $CD3^{++}$, apparently mature cells, with a reduction of the proportion of $CD3^-$ cells compared to free thymocytes. The few $CD4^-CD8^+$ thymocytes associated with the adherent M-ROS were predominantly $CD3^-$ immature cells, as expected for a cortical population. However, the majority of the more prominent $CD4^+CD8^-$ thymocytes from M-ROS were $CD3^{++}$, apparently mature cells, a surprising result for a putative cortical population. Similar findings were obtained with C57BL/6 mice (results not shown), the additional CD3 marker providing a clear delineation of the group of $CD4^+CD8^-CD3^{++}$ cells associated with M-ROS, despite the higher level of $CD4^+CD8^+$ cells.

### Do the Mature Single Positive Thymocytes of ROS Differ from Mature Medullary Thymocytes?

The majority of thymocytes associated with ROS were of the $CD4^+CD8^+$ cortical phenotype in accordance with the view that M-ROS, if not D-ROS, are mainly derived from the cortex. It was therefore surprising to find any mature $CD4^+CD8^-CD3^{++}$ or $CD4^-CD8^+CD3^{++}$ thymocytes associated with ROS, since cells of this pheno-

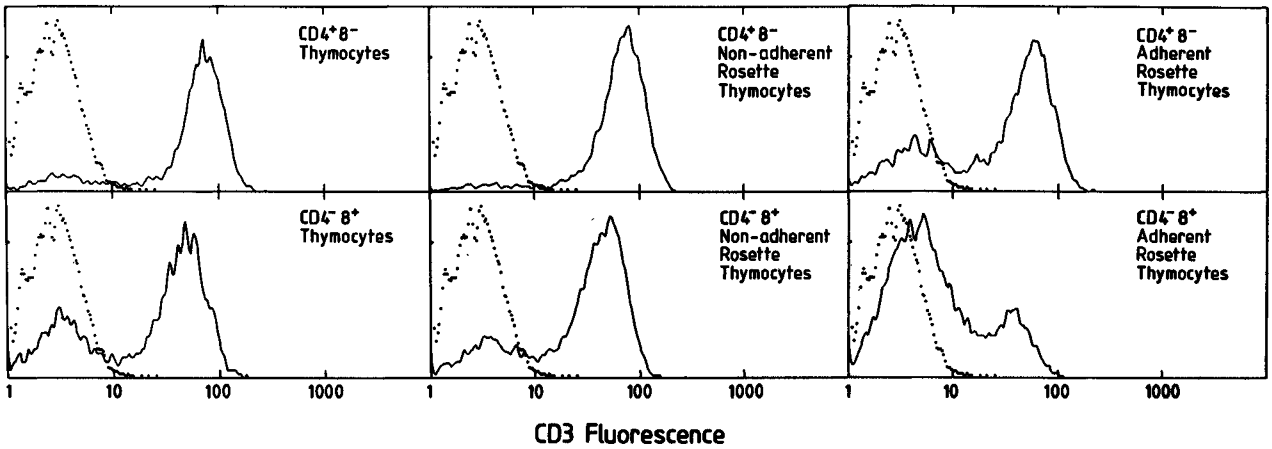

FIGURE 4 The expression of CD3 by the $CD4^-CD8^+$ and the $CD4^+CD8^-$ populations obtained from ROS. D-ROS were separated as nonadherent rosettes, and M-ROS as adherent rosettes. Cells were stained with anti-CD4, anti-CD8, and anti-CD3, and the levels of CD3 expression plotted for the gated $CD4^-CD8^+$ or $CD4^+CD8^-$ populations. Broken lines give the background, omitting the biotinylated anti-CD3 reagent. Note that relatively few adherent rosette thymocytes were $CD4^-CD8^+$.

type are predominantly localized in the medulla (Shortman et al., 1985, 1987). To check if the CD3++ single positives of ROS were mature by all surface markers, they were analyzed for H-2K expression, since this is high on mature peripheral T cells or mature medullary thymocytes, but very low on CD4+CD8+ cortical thymocytes (Scollay and Shortman, 1983). As shown in Fig. 5,

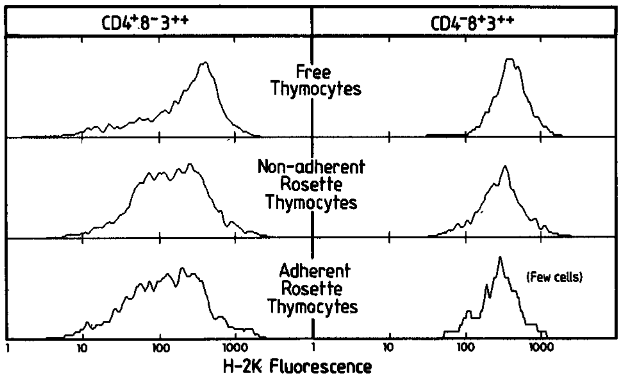

FIGURE 5 The level of H-2K on the surface of CD4-CD8+CD3++ and CD4+CD8-CD3++ thymocytes from D-ROS and M-ROS. Cells dissociated from ROS were stained in four fluorescent colors with anti-CD4, anti-CD8, anti-CD3, and anti-H-2K, and the H-2K distribution on the subpopulations obtained by gating. The single positives from D-ROS (nonadherent) and M-ROS (adherent) were compared with those from a mechanically dissociated free-thymocyte preparation. Very few CD4-CD8+CD3++ cells were obtained from the adherent rosettes, as expected from Figs. 2 and 3, but the H-2K distribution of these few is presented. The background fluorescence peaked around a fluorescence value of 5, so virtually all cells showed some H-2K expression.

the majority of CD4-CD8+CD3++ and CD4+CD8-CD3++ thymocytes associated with both M-ROS and D-ROS expressed lower levels of H-2K than found on free single positive thymocytes. This effect was most marked with ROS-associated CD4+CD8-CD3++ thymocytes, which included cells expressing all H-2K levels between that of cortical CD4+CD8+ cells and that of free mature CD4+CD8- thymocytes. This suggested the ROS-associated "mature" thymocytes were recently derived from cortical cells and had not fully completed the transition to a mature surface phenotype. Note that even the free CD4+CD8-CD3++ thymocyte population included a few cells expressing low levels of H-2K, these presumably representing "almost mature" cells.

### Are CD4+CD8+CD3++ Developmental Intermediates Associated with ROS?

Since the single positive mature thymocytes of ROS appeared to have recently matured from CD4+CD8+ cortical thymocytes, we checked for the presence in ROS of thymocytes representing still earlier transit stages. One such intermediate is the minor CD4+CD8+ subpopulation expressing high levels of TcR-CD3, first noted in the human thymic cortex (Blue et al., 1987), and recently shown by us and others studying the murine thymus to be a postselection intermediate en route to mature T cells (Guidos et al., 1990; Hugo, Boyd, Waanders, Petrie, and Scollay, submitted for publication; Shortman, Vremec, and Egerton, submitted for publication). Additional transit stages can be seen if total free thymocytes are stained with CD4, CD8, and CD3, then the CD3++ cells analyzed by gating; a "boomerang"-shaped distribution is then obtained, with the CD4+CD8+CD3++ intermediate as the starting point, extending out to the mature single positives as the developing cells lose either CD4 or CD8 (Fig. 6). When total ROS-associated thymocytes were analyzed in this way, all these intermediate forms were enriched in the ROS structures (Fig. 6). When M-ROS and D-ROS were separated, both contained CD4+CD8+CD3++ intermediates (Fig. 6). However, M-ROS showed only one arm of the "boomerang" distribution of CD3+ cells, that leading to the CD4+CD8-CD3++ population. In contrast, both sets of intermediates were seen associated with D-ROS.

It was important to establish that the "triple positive" (CD4+CD8+CD3++) cells in the ROS were not aggregates of cells or residual undissociated ROS. Various regions of the CD4 and CD8 distribution of the ROS-associated CD3++ cells were analyzed for size by low-angle light scatter, and also sorted and examined by phase-contrast light microscopy. A very minor subgroup of "cells" expressing very high levels of CD4 and CD8 (visible in Fig. 6) did give exceptionally high light-scatter values, and when sorted, these were seen to be mainly ROS stromal cells with several residual thymocytes attached. This area could readily be gated out of analyses. However, the main CD4+CD8+CD3++ population from ROS (with slightly lower levels of CD4 and CD8 than most CD4+CD8+ thymocytes) gave only marginally greater light-scatter values than typical

CD4$^+$CD8$^+$ cortical thymocytes, and on sorting and microscope examination, these were all single cells. The CD3$^{++}$ single positives of ROS had light-scatter characteristics similar to those of medium-sized mature thymocytes, as expected. It was concluded that the vast majority of the CD4$^+$CD8$^+$CD3$^{++}$ entities of ROS were individual cells and not aggregation artefacts, and that ROS concentrated a selection of cells at intermediate stages of maturation.

### DISCUSSION

These studies demonstrate that the stromal cells of thymic rosettes associate with particular subpopulations of developing thymocytes. Although our previous analysis of the thymocytes of total ROS revealed few differences from the total free thymocytes, this was largely due to a failure to segregate the different types of ROS. By separating M-ROS and D-ROS, differences in associated thymocytes became apparent. These differences might reflect a selectivity in the binding mechanism, macrophages having a different pattern of adhesion molecules from dendritic cells. Alternatively, the binding might be nonselective, the differences in composition simply reflecting the free thymocyte population balance in different regions of the thymus, with M-ROS sampling an outer cortical region, D-ROS sampling the corticomedullary junction region. The finding that the composition of M-ROS differed from that of the 5% of thymocytes selectively labeled by transcapsular administration of a dye argues for selectivity in binding. However, it may be that M-ROS simply sample a region further within the thymus than the subcapsular zone.

Some of our findings on the thymocyte composition of the two types of ROS fit with current perceptions of their biological role. Dendritic cells are believed to be involved in antigen presentation and negative selection, a relatively late stage of T-cell development within the thymus. The concentration in D-ROS of thymocytes expressing high levels of the TcR-CD3 complex is in accordance with this role, and agrees with the results of Kyewski et al. (1989). However, the

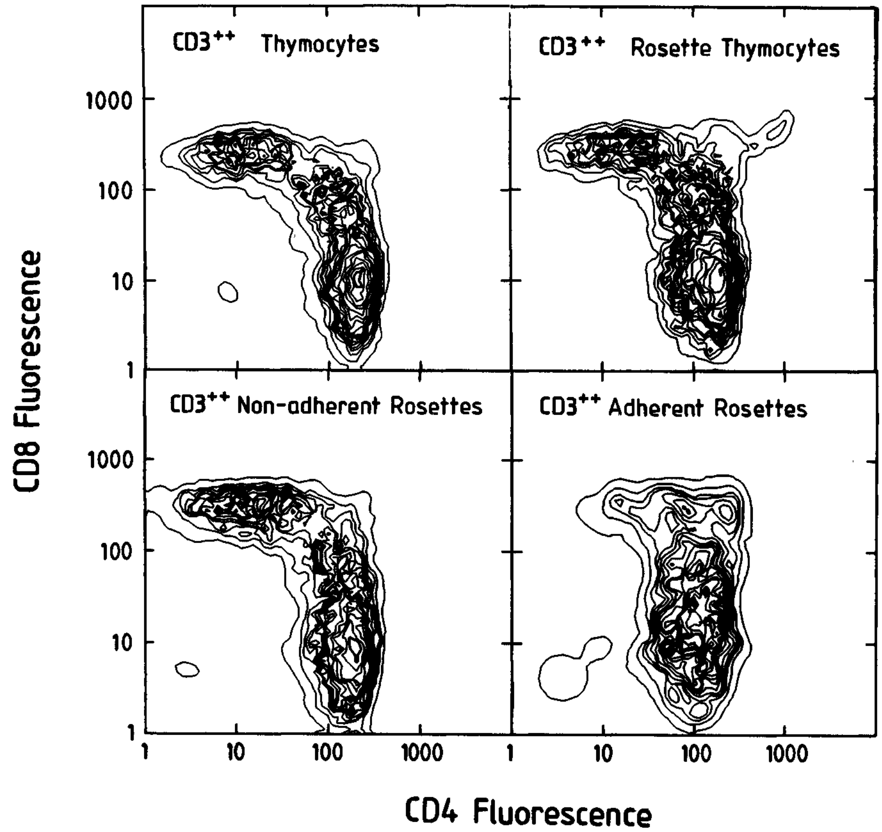

FIGURE 6 The level of CD4$^+$CD8$^+$CD3$^{++}$ thymocytes and other late developmental intermediates in ROS. The dissociated ROS were stained with anti-CD4, anti-CD8, and anti-CD3, and the levels of CD4 and CD8 expression on cells gated for high levels of CD3 (CD3$^{++}$) then analyzed. The comparison of free thymocytes and total ROS was a separate experiment from the comparison of D-ROS (non-adherent) and M-ROS (adherent).

binding of thymocytes to D-ROS is clearly not by the TcR alone, since D-ROS contained a proportion of TcR-CD3$^-$ cells. An association between the outer cortical macrophages of M-ROS and early thymocytes has been found by Kyewski (1987) to be the first demonstrable stromal cell–lymphoid cell interaction after a stem cell seeds the thymus. The relatively high incidence in M-ROS of CD4$^-$CD8$^-$ and CD4$^+$CD8$^+$ thymocytes, and the low incidence of cells expressing high levels of TcR-CD3, is in accordance with a role in the early phase of T-cell development.

A more puzzling observation has been the association of a definite proportion of apparently mature CD4$^-$CD8$^+$CD3$^{++}$ and CD4$^+$CD8$^-$CD3$^{++}$ thymocytes with ROS (Shortman et al., 1989). Our previous studies suggested these represented a preexistent association within the thymus, rather than a random attachment after disruption of the organ (Shortman et al., 1989). The proposed corticomedullary junction localization of D-ROS could explain how the one structure includes both cells typical of the cortex (CD4$^+$CD8$^+$) and cells typical of the medulla (CD4$^-$CD8$^+$CD3$^{++}$ and CD4$^+$CD8$^-$CD3$^{++}$), but the existence of a significant proportion of CD4$^+$CD8$^-$CD3$^{++}$ cells in the supposedly outer cortical M-ROS presented a paradox. The level of H-2K on these "mature" ROS-associated cells now suggests they are different from the free mature thymocytes of the medulla, and that they may be of recent origin from the cortical CD4$^+$CD8$^+$ population. The presence of a high proportion of developmental intermediates associated with ROS, and in particular the CD4$^+$CD8$^+$CD3$^{++}$ population believed to be the immediate precursor of mature cells, adds weight to this view. We propose that the mature thymocytes of ROS are recently formed cells, associating with the stromal cells soon after their formation in the cortex, prior to their entry into the final pool of mature cells in the medulla.

The striking selective association with M-ROS of CD4$^+$CD8$^-$CD3$^{++}$ thymocytes, but not CD4$^-$CD8$^+$CD3$^{++}$ thymocytes, requires an additional explanation. It may be that the environment of the thymus where M-ROS are located is selective for the maturation of the CD4$^+$CD8$^-$, class II MHC reactive T cells and that development of the CD4$^-$CD8$^+$ class I MHC reactive T cells occurs elsewhere. An alternative explanation is that the selective events eventually leading to the production of both classes of mature T cells occur about the same time and in the same region of the thymus, but that it then takes much longer to downregulate CD4 than CD8. The early stages of the CD4$^-$CD8$^+$ lineage would then be present in M-ROS, but be amongst the CD4$^+$CD8$^+$CD3$^{++}$ thymocytes, and final downregulation of CD4 would occur only after the cells leave the M-ROS and move toward the corticomedullary junction. Some evidence in support of this latter model comes from our recent kinetic studies on the terminal stages of production of mature thymocytes from CD4$^+$CD8$^+$ cortical cells (Shortman, Vremec, Egerton, submitted for publication): upregulation of TcR-CD3 takes about 1 day, but downregulation of the other cortical markers takes about 3 days more, with loss of CD8 preceding loss of CD4.

What is the significance of these associations of intermediates in the late stages of the T-cell maturation process with ROS? It might represent no more than a pickup by the stromal cells of a random sample of cells from a particular thymic microenvironment, as discussed previously. It is, however, of more interest to propose the associations have an immunological function, and experiments involving treatment of mice with antibodies against the TcR-CD3 complex encourages this view (Kyewski et al., 1989). The complexes might represent sites of positive selection of the TcR repertoire. Alternatively, the positively selected, maturing T cells might be bound to stromal cells as part of a negative selection system for deleting autoreactive cells. To test these possibilities, experiments are underway to determine if the TcR-bearing thymocytes of ROS differ from free thymocytes in their representation of different TcR V$\beta$ regions.

### MATERIALS AND METHODS

### Animals

Specific pathogen-free 5-week-old female mice of either the CBA CaH Wehi or the C57BL/6J Wehi strains were used, bred at The Walter and Eliza Hall Institute animal facility. The mice had been left undisturbed and without cage changes for 2 weeks before use.

### Preparation of Thymic Rosettes (ROS)

The isolation of ROS from the thymus and their purification by unit-gravity elutriation has been described in detail elsewhere (Andrews and Shortman, 1985; Shortman et al., 1989). Briefly, 12 thymuses were pooled, cut into fragments, and most free thymocytes were released mechanically. The stromal fragments remaining were digested at room temperature with collagenase (containing DNAse to reduce clumping). The digest was immediately placed in a conical chamber at 4°C, and medium, stabilized by a FCS gradient, pumped in underneath, at a rate sufficient to separate free cells from ROS from larger aggregates as the preparation was elutriated from the chamber. In the present studies, the elutriation medium was changed to one based on mouse osmolarity, Hepes-buffered RPMI-1640, in view of the subsequent steps involving short periods of incubation at 37°C; the FCS gradient ranged from 20 to 60%. Fractions containing the larger ROS (7–20 thymocytes attached to a central stromal cell) were pooled. These pooled fractions included 1–3 free mononuclear cells per ROS; some of these free cells had been released from the ROS themselves, some were lymphoid and nonlymphoid contaminants. The level and effect of non-ROS contaminants has been considered in detail previously (Shortman et al., 1989).

### Separation of M-ROS and D-ROS by Adherence

The two types of ROS were generally separated on the basis of differences in their adherence properties (Kyewski et al., 1987). In our final procedure, no concentration by centrifugation was used prior to the adherence step, to avoid the possibility of cell exchange between rosettes. The 70 ml pooled fractions containing highly purified ROS in culture medium and FCS were placed in large flat-bottomed vessels (glass or plastic petri dishes or flasks) so the depth of medium was less than 0.5 cm, then incubated at 37°C for 50–60 min in a humidified 10% $CO_2$-in-air atmosphere. During this time, the ROS settled onto the flat surface, and a proportion adhered strongly. Nonadherent ROS were resuspended by gentle agitation, and the supernatant removed. The adherent cells were gently washed with prewarmed, preequilibrated medium, and the washings combined with the original supernatant to give the nonadherent D-ROS fraction. These were concentrated by centrifugation to a pellet, then resuspended and incubated with occasional vigorous mixing for 4 min at 37°C in a $Ca^{++}$ and $Mg^{++}$-free, EDTA-containing medium to dissociate the rosettes, as described previously (Shortman et al., 1989). The same $Ca^{++}$ and $Mg^{++}$ free, EDTA-containing medium was used to treat (4 min, 37°C) the adherent M-ROS attached to the flat base of the vessels, to dissociate the ROS in situ. Even with vigorous mixing, many of the central macrophages remained attached to the surface, but the associated thymocytes were released and recovered. Cells from both ROS fractions were recovered by layering over, then spinning through, a zone of FCS; however, from this point onwards, all such FCS preparations contained sufficient EDTA to chelate all $Ca^{++}$ and $Mg^{++}$, to prevent reformation of rosettes in the cell pellets.

### Separation of M-ROS and D-ROS with Anti-MAC-1 and Magnetic Beads

The ROS in the pooled fractions were concentrated by low-speed centrifugation, gently suspended, then treated with anti-MAC-1 (M1/70.15) (Springer et al., 1978) for 20 min at 4°C, as in a staining procedure. The suspension was washed and excess antibody removed by layering over FCS (not containing EDTA), one low-speed centrifugation, and removal of the supernatant. The ROS were gently resuspended, mixed with anti rat Ig-coated magnetic beads (Dynabeads, Dynal, Oslo, Norway) at a ratio of approximately 7 beads per ROS cell, and held at 4°C for 20 min with occasional gentle mixing. The beads and bound M-ROS were then separated with a strong magnet. The unbound D-ROS were recovered from the supernatant by centrifugation and dissociated by EDTA treatment, as before. The M-ROS were dissociated by direct EDTA treatment of the separated slurry of beads and bound ROS, and the beads with bound macrophages then removed from the EDTA-containing medium using a strong magnet. Thymocytes dissociated from the D-ROS and M-ROS were then treated as before.

### Immunofluorescent Staining and Flow-Cytometric Analysis

Full details of the monoclonal antibodies and fluorescent reagents, the staining protocols and the flow cytometry have been given previously (Shortman et al., 1988, 1989; Wilson et al., 1988). The main monoclonal antibodies used were anti-CD4, GK1.5 (Dialynas et al., 1983); anti-CD8, 53-6.7 (Ledbetter and Herzenberg, 1979); anti-CD3, KT3-1.1 (Tomonari, 1988); anti-H-$2K^k$, 11-4.1 (Oi et al., 1978); and anti-HSA, M1/69 (Springer et al., 1978). Hybridomas were grown in this laboratory and the antibodies purified and directly conjugated to FITC, to allophycocyanin, or to biotin for use with PE-avidin or Texas Red-streptavidin second stage. After each staining period (20 min at 0–4°C), cells were washed by dilution in medium, then layering over, and spinning through FCS. The FCS contained sufficient added EDTA to chelate divalent metals and so prevent reformation of rosettes. Propidium iodide was included in the final wash medium to stain dead cells. Immediately before analysis, cells were passed through a 25-gauge needle to disrupt any aggregates. Two-, three-, or four-color flow-cytometric analysis was performed using a FACStar Plus or a modified FACS II instrument (Becton Dickinson, Mountain View, CA). Dead cells and debris, and very large nonlymphoid cells and cell aggregates, were excluded on the basis of low-angle and side scatter, and of very bright red propidium-iodide fluorescence.

### Transcapsular Labeling for Intrathymic Localization

The procedure of Scollay et al. (1980), modified for use with the blue fluorescent DNA stain Hoechst 33342 (Calbiochem-Behring), was used as described elsewhere (Shortman et al., 1985). Briefly, intact thymuses were immersed in Hoechst 33342 solution (100 $\mu$g/ml in balanced salt solution containing 5% FCS) for 30 min at 37°C. The thymuses were washed in cold medium, and then M-ROS and D-ROS were isolated as before, or, alternatively, free thymocyte suspensions were prepared by a conventional mechanical disruption procedure. The free cells or cells from dissociated ROS were analyzed by flow cytometry on the modified FACS II instrument (Shortman et al., 1985). Under these conditions, 2–10% of all free thymocytes could be classified as blue fluorescent, depending on the gates used.

### ACKNOWLEDGEMENTS

This work was supported by the National Institutes of Health, USA, Grant Number AI 17310; by the C.H. Warman Research Fund; and by the National Health and Medical Research Council, Australia. We thank Roland Scollay, Patrice Hugo, and Howard Petrie for discussion and advice.

*(Received December 21, 1990)*

*(Accepted January 10, 1991)*

### REFERENCES

Andrews P., and Shortman K. (1985). Zonal unit-gravity elutriation: a new technique for separating large cells and multicellular complexes from cell suspensions. Cell Biophysics **7**: 252–266.

Blue M.-L., Daley J.F., Levine H., Craig K.A., and Schlossman S.F. (1987). Identification and isolation of a $T4^+T8^+$ cell with high T3 expression in human thymus: a possible late intermediate in thymocyte differentiation. J. Immunol. **139**: 1065–1069.

Crispe I.N., and Bevan M.J. (1987). Expression and functional significance of the J11d marker on mouse thymocytes. J. Immunol. **138**: 2013–2018.

Dialynas D.P., Wilde D.B., Marrack P., Pierres A., Wall K.A., Havran W., Otten G., Loken M.R., Pierres M., Kappler J., and Fitch F.W. (1983). Characterization of the murine antigenic determinant, designated L3T4a, recognized by monoclonal antibody GK1.5: expression of L3T4a by functional T cell clones appears to correlate primarily with class II MHC reactivity. Immunol. Rev. **74**: 29–56.

Guidos C.J., Danska J.S., Fathman C.G., and Weissman I.L. (1990). T cell receptor-mediated negative selection of autoreactive T lymphocyte precursors occurs after commitment to the CD4 or CD8 lineages. J. Exp. Med. **172**: 835–845.

Hugo P., Waanders G.A., Scollay R., Shortman K., and Boyd R. (1990). Ontogeny of a novel $CD4^+CD8^-CD3^-$ thymocyte subpopulation: a comparison with $CD4^-CD8^+CD3^-$ thymocytes. Int. Immunol. **2**: 209–218.

Kyewski B.A. (1987). Seeding of thymic microenvironments defined by distinct thymocyte-stromal cell interactions is developmentally controlled. J. Exp. Med. **166**: 520–538.

Kyewski B.A., Momburg F., and Schirrmacher V. (1987). Phenotype of stromal cell-associated thymocytes in situ is compatible with selection of the T cell repertoire at an "immature" stage of thymic T cell differentiation. Eur. J. Immunol. **17**: 961–967.

Kyewski B.A., Rouse R.V., and Kaplan H.S. (1982). Thymocyte rosettes: multicellular complexes of lymphocytes and bone marrow-derived stromal cells in the mouse thymus. Proc. Natl. Acad. Sci. USA **79**: 5646–5650.

Kyewski B.A., Schirrmacher V., and Allison J.P. (1989). Antibodies against the T cell receptor/CD3 complex interfere with distinct intra-thymic cell-cell interactions in vivo: correlation with arrest of T cell differentiation. Eur. J. Immunol. **19**: 857–863.

Ledbetter J.A., and Herzenberg L.A. (1979). Xenogeneic monoclonal antibodies to mouse lymphoid differentiation antigens. Immunol. Rev. **47**: 63–90.

MacDonald H.R., Budd R.C., and Howe R.C. (1988). A $CD3^-$ subset of $CD4^-8^+$ thymocytes: a rapidly cycling intermediate in the generation of $CD4^+8^+$ cells. Eur. J. Immunol. **18**: 519–523.

Matsumoto K., Yoshika Y., Matsuzaki G., Asano T., and Nomoto K. (1989). A novel $CD3^-$ $J11d^+$ subset of $CD4^+CD8^-$ cells repopulating thymus in radiation bone marrow chimeras. Eur. J. Immunol. **19**: 1203–1208.

Oi V., Jones P.P., Goding J.W., Herzenberg L.A., and Herzenberg, L.A. (1978). Properties of monoclonal antibodies to mouse Ig allotypes, H-2 and Ia antigens. Cur. Top. Microbiol. Immunol. **81**: 115–129.

Scollay R., Jacobs S., Jerabek L., Butcher E., and Weissman I.L. (1980). T cell maturation: thymocyte and thymus migrant subpopulations defined with monoclonal antibodies to MHC region antigens. J. Immunol. **124**: 2845–2853.

Scollay R., and Shortman K. (1983). Thymocyte subpopulations: an experimental review, including flow cytometric cross-correlations between the major murine thymocyte markers. Thymus **5**: 245–295.

Scollay R., and Shortman K. (1985). Identification of early stages of T lymphocyte development in the thymus cortex and medulla. J. Immunol. **134**: 3632–3642.

Shortman K., Mandel T., Andrews P., and Scollay R. (1985). Are any functionally mature cells of medullary phenotype located in the thymus cortex? Cell. Immunol. **93**: 350–363.

Shortman K., Vremec D., D'Amico A., Battye F., and Boyd R. (1989). Nature of the thymocytes associated with dendritic cells and macrophages in thymic rosettes. Cell. Immunol. **119**: 85–100.

Shortman K., Wilson A., Egerton M., Pearse M., and Scollay R. (1988). Immature $CD4^-CD8^+$ murine thymocytes. Cell. Immunol. **113**: 462–479.

Shortman K., Wilson A., van Ewijk W., and Scollay R. (1987). Phenotype and localization of thymocytes expressing the homing receptor associated antigen MEL-14: arguments for the view that most mature thymocytes are located in the medulla. J. Immunol. **138**: 342–351.

Springer T., Galfre G., Secher D.S. and Milstein C. (1978). Monoclonal xenogeneic antibodies to murine cell surface antigens: identification of novel leukocyte differentiation antigens. Eur. J. Immunol. **8**: 539–551.

Tomonari K. (1988). A rat antibody against a structure functionally related to the mouse T-cell receptor/T3 complex. Immunogenetics **28**: 455-458.

Wilson A., D'Amico A., Ewing T., Scollay R., and Shortman K. (1988). Subpopulations of early thymocytes: a cross-correlation flow-cytometric analysis of adult mouse Ly $2^-$ $L3T4^-$ ($CD8^-CD4^-$) thymocytes using eight different surface markers. J. Immunol. **140**: 1461–1469.

